# Zonulin, inflammation and iron status in patients with early stages of chronic kidney disease

**DOI:** 10.1007/s11255-017-1741-5

**Published:** 2017-11-13

**Authors:** Ewelina Lukaszyk, Mateusz Lukaszyk, Ewa Koc-Zorawska, Anna Bodzenta-Lukaszyk, Jolanta Malyszko

**Affiliations:** 1 2nd Department of Nephrology and Hypertension with Dialysis Unit, Medical University of Bialystok, Marii Skłodowskiej-Curie 24a, 15-276 Białystok, Poland; 2 Department of Allergy and Internal Medicine, Medical University of Bialystok, Białystok, Poland

**Keywords:** Zonulin, Iron, Inflammation, Chronic kidney disease, Hepcidin

## Abstract

**Background/aims:**

Zonulin is the only known regulator of intestinal permeability. It is also considered as a potential inflammatory marker in several conditions such as diabetes and inflammatory bowel syndrome. The aim of the study was to investigate zonulin levels in patients with early stages of CKD and its possible correlation with inflammation, anemia and iron status parameters.

**Methods:**

Eighty-eight patients with early stages of CKD and 23 healthy volunteers were enrolled in the study. Zonulin, hepcidin-25, soluble transferrin receptor, interleukin-6 and high-sensitivity C-reactive protein were measured using commercially available assays.

**Results:**

Zonulin was significantly lower among patients with CKD in comparison with healthy volunteers. There were no statistically significant differences in zonulin concentration between patients with and without inflammation. Zonulin was significantly correlated with hepcidin only in patients with inflammation. Zonulin was neither related to iron nor related to ferritin.

**Conclusions:**

Zonulin cannot be considered as an inflammatory marker in CKD. It does not play a role in the disturbances of iron metabolism in CKD. Its physiological role remains to be elucidated.

## Introduction

Inflammation is one of the pathomechanisms responsible for progressive impairment in kidney function and is associated with common complications in chronic kidney disease (CKD) such as anemia. Inflammation also affects iron metabolism and is responsible for deranged iron distribution despite adequate iron stores in the body. Iron absorption takes place in the small intestine, where zonulin is the only known modulator of intracellular tight junction [1]. Zonulin has been identified as pre-haptoglobin 2 and regarded only as an inactive precursor for haptoglobin 2 (HP2). Zonulin is also considered as a potential inflammatory marker [2]. Intestinal permeability and zonulin disturbances have been reported to be associated with diabetes type 1 [3], type 2 [4, 5], inflammatory bowel syndrome [6] and other conditions with low-grade inflammation [7].

In our previous study on small group of patients in second and third stage of CKD, we observed correlations of zonulin with interleukin-6 (IL-6) and hemojuvelin [8]. Therefore, we aimed to study zonulin levels in a larger population of patients with early stages of CKD and investigate its possible correlation with inflammation, anemia and iron status.

## Patients and methods

Eighty-eight patients with CKD (defined as: an estimated glomerular filtration rate (eGFR) ≤ 60/ml/min/1.75 m^2^ or presence of hematuria or proteinuria for ≥ 3 month) were enrolled into the study and divided into two groups—with and without subclinical inflammation according to hsCRP measurements. Zonulin concentration was also measured in the healthy volunteers (*n* = 23). Patients with clinically evident inflammation, acute thrombosis, active malignancy, acute cardiovascular complications (including uncontrolled hypertension, acute coronary syndrome and acute heart failure), anemia and/or iron deficiency treatment, blood transfusions within 3 months prior the study and immunosuppressive therapy were excluded from the study. Every subject gave informed consent, and the study protocol was approved by the Medical University Ethics Committee.

Medical history (including demographic characteristics as well as current pharmacotherapy) and blood samples were collected in all subjects at the time of enrollment in the morning after an overnight fast. Hematological measurements were taken using fresh venous blood with EDTA and clotted blood. The plasma and serum were centrifuged and frozen at − 70 °C until further laboratory analysis.

Serum hemoglobin, creatinine, iron, total iron binding capacity (TIBC) and ferritin levels were obtained using standard laboratory methods (automated system) in certified local central laboratory. Transferrin saturation (TSAT) was calculated as the ratio of serum iron and TIBC and expressed as a percentage. Glomerular filtration rate was estimated with the use of Chronic Kidney Disease Epidemiology Collaboration formula (CKD-EPI).

Zonulin was assessed using kit from Immundiagnostik, Germany. Hepcidin-25 was measured using an assay from Bachem, UK. Soluble transferrin receptor—sTfR and interleukin-6 were studied by using kits from R&D Systems, UK. High-sensitivity C-reactive protein—hsCRP was measured with the use of CRP ELISA Kit LDN Labor Diagnostika Nord GmbH&Co KG, Germany.

Data with normal distribution were reported as mean ± standard deviation, and non-normally distributed as median and interquartile range. Variables with skewed distribution were log (ln)-transformed before further statistical analysis. Analyses of the correlation of each parameter were performed using Pearson or Spearman correlation coefficients. The multiple regression analysis was used to determine independent factors affecting the dependent variables. Factors showing linear correlation with zonulin or hepcidin (*p* < 0.05) were included in the analysis. All statistical analyses were performed using Statistica 12.5 (StatSoft) computer software.

## Results

**Fig. 1:**
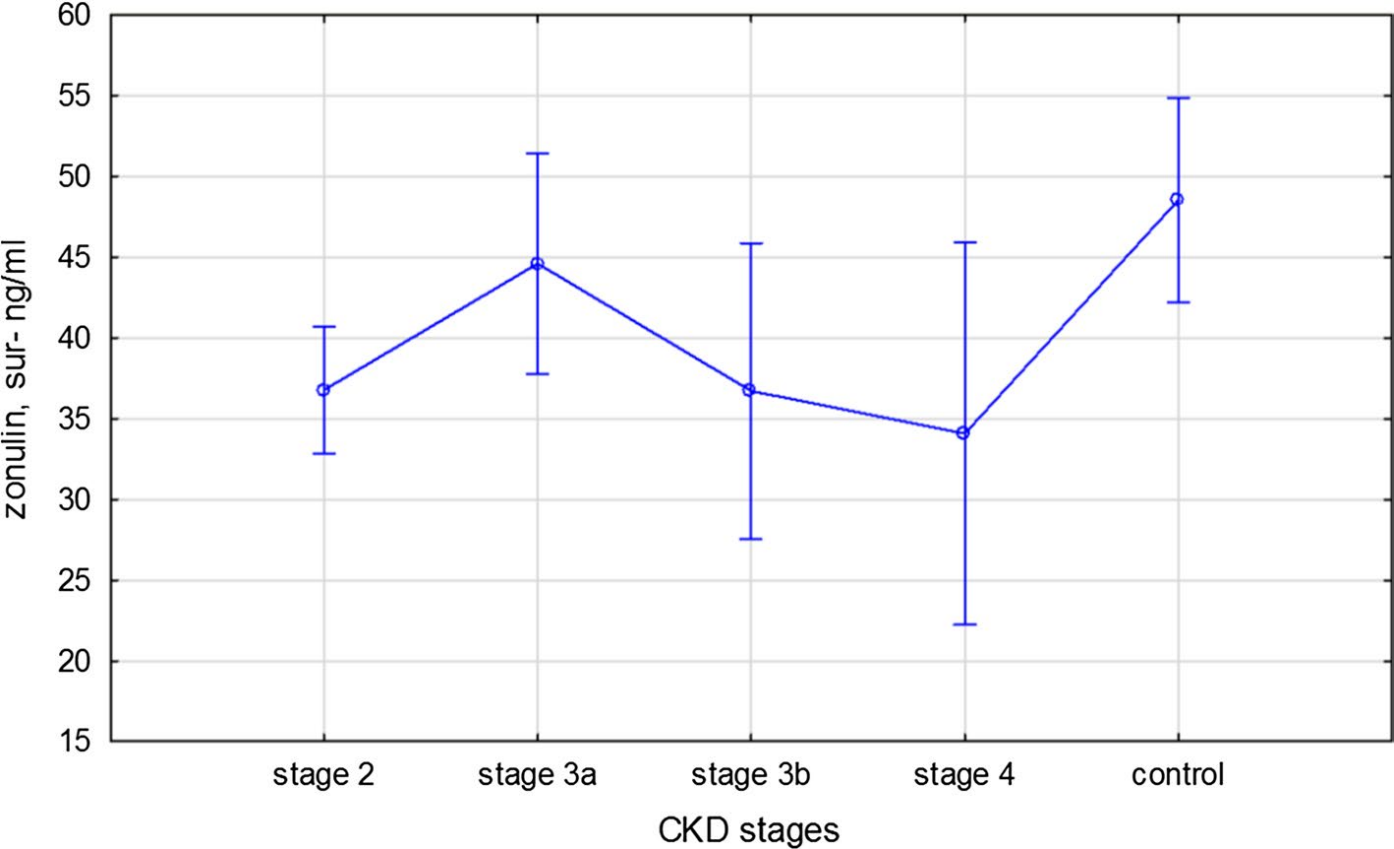
Zonulin concentrations in CKD patients. One-way ANOVA, F(3, 84) = 0.62 *p* = 0.85 vs control group* F*(4,1050) = 3.26, *p* = 0.014

Patients’ laboratory characteristic is presented in Table 1. Zonulin was significantly lower in patients with CKD than in healthy volunteers (38.2 ± 16 vs 48.5 ± 6.4 ng/mL; *p* = 0.003). There is a trend toward decrease in zonulin levels with the severity of CKD (Fig. 1). There were no statistically significant differences in zonulin concentration between patients with and without inflammation as well as anemic and non-anemic patients with CKD. Patients with inflammation had significantly lower hemoglobin, iron and TSAT values. Hepcidin concentration was higher in patients with inflammation.

**Table 1:** Characteristic of patients with chronic kidney disease and healthy control

	CKD patients with inflammation hsCRP > 10 mg/dL *n* = 35	CKD patients with no inflammation hsCRP < 10 mg/dL *n* = 53	Control *n* = 23	*p* value (inflammation vs no inflammation)	*p* value (CKD with no inflammation vs control)
Age, years	73.9 ± 10.9	68 ± 11.3	52 ± 9.0	0.02	< 0.001
Hemoglobin, g/dL	12.2 ± 2.1	13.7 ± 2.0	14 ± 1.0	< 0.001	0.5
Iron, μg/dL	54.2 ± 28.0	86.9 ± 35.2	95.7 ± 25	< 0.001	0.28
TSAT, %	23.2 ± 12.1	30.3 ± 13.4	29.1 ± 7	0.01	0.69
Ferritin, mg/dL	171.0 (75.6–339.8)	123.1 (90.3–190.9)	104 61–159)	0.2	0.1
eGFR, mL/min/1.73 m^2^	60.8 ± 20.1	66.2 ± 18.1	98.1 ± 16	0.2	< 0.001
IL-6, pg/mL	4.1 (1.04–12.2)	0.8 (0.22–3.4)	0.5 (0.1–0.9)	< 0.001	0.2
Hepcidin-25, ng/mL	41.7 (25.1–87.9)	35.4 (18.2–47.4)	24.5 (19.1–33)	0.04	0.36
Zonulin, ng/mL	36.6 ± 20.6	37.3 ± 12.1	48.5 ± 6.4	0.84	< 0.001
sTfR, nmol/L	19.0 (16.4–26.1)	17.1 (14.4–23.3)	10 (8–11)	0.13	0.02
hsCRP, mg/dL	25.2 (18.9–40.9)	3.9 (1.6–5.9)	0.9 (0.3–1.4)	< 0.000	0.01

**Fig. 2:**
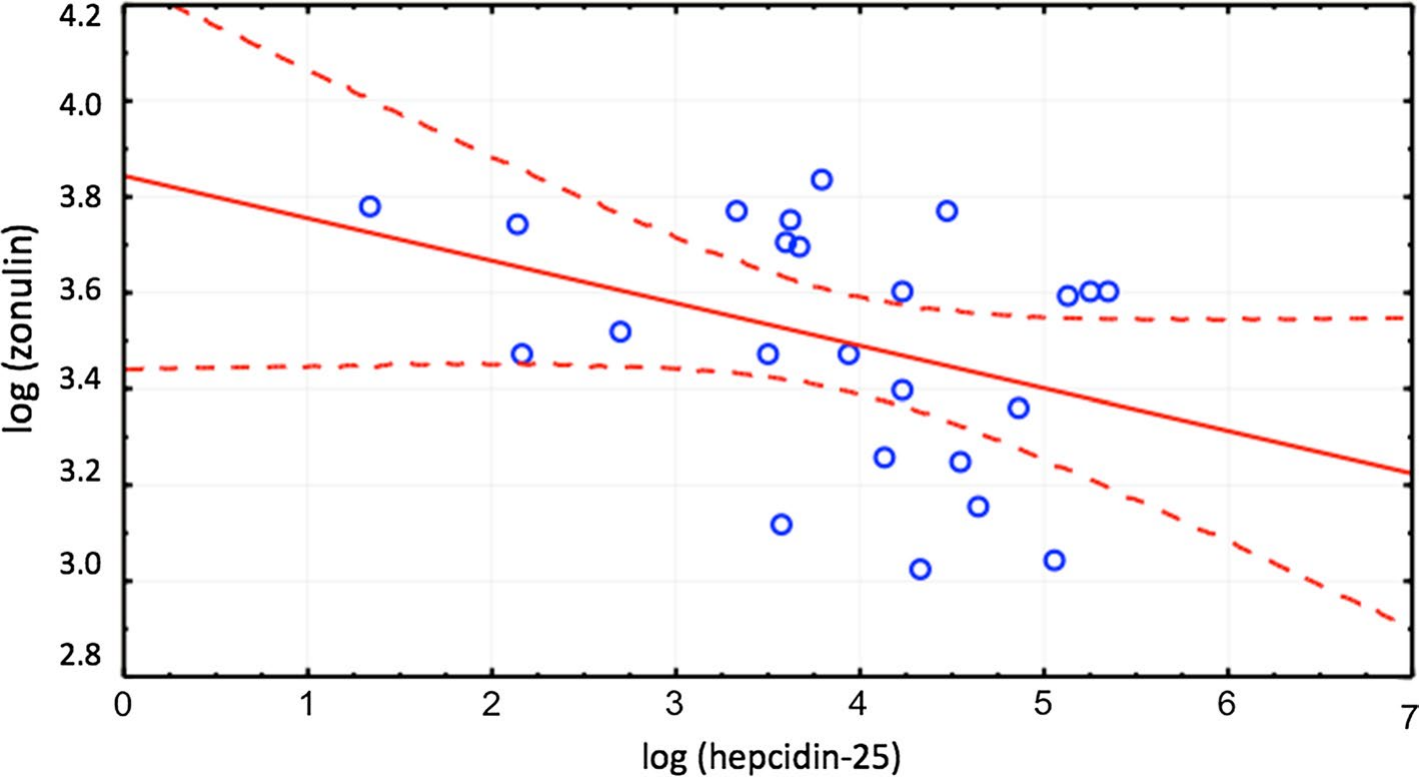
Correlation between zonulin and hepcidin in patients with CKD and inflammation, *r* = − 0.37

**Fig. 3:**
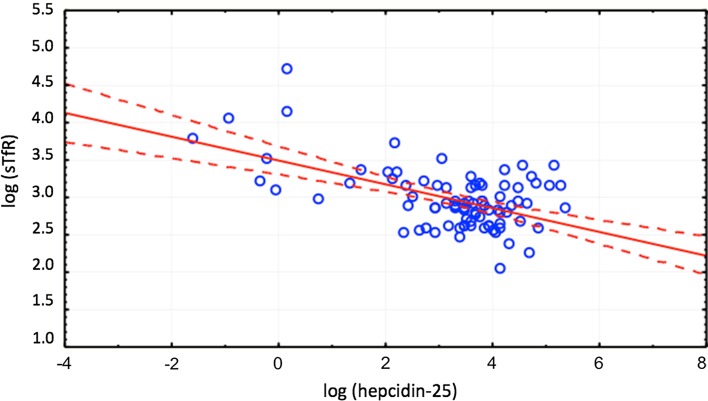
Correlation between hepcidin-25 and sTfR in patients with CKD, *r* = − 0.41

Zonulin was significantly correlated with hepcidin (*r* = − 0.37, *p* < 0.05) only in patients with inflammation defined as elevated hsCRP > 10 mg/dL (Fig. 2). Hepcidin was correlated with TSAT (*r* = 0.49, *p* < 0.05), ferritin (*r* = 0.73, *p* < 0.05) and sTfR (*r* = − 0.41, *p* < 0.05—Fig. 3) in both groups as well as in independent analysis. Zonulin was not related to iron or ferritin.

## Discussion

In this study, we found that zonulin correlated significantly with hepcidin in patients with early stages of CKD and inflammation defined as elevated hsCRP > 10 mg/dL. There were no differences in zonulin concentration between anemic and non-anemic patients with CKD as well as patients with and without inflammation. We did not find any significant correlations between zonulin and iron status parameters.

Zonulin is known as a mediator regulating intestinal permeability throughout disassembling intracellular tight junctions [1]. In recent years, tight junctions and zonulin begin to emerge in a spotlight of many areas of medicine including autoimmune diseases, malignancy and disorders of central nervous system [9]. In addition, zonulin overexpression in the immune-mediated diseases such as diabetes mellitus type 1 and celiac disease [10, 11] suggests that zonulin is an inflammatory marker, like its precursor, haptoglobin [4], a liver acute-phase response protein. Moreover, the expression of haptoglobin in hepatocytes is increased by number of proinflammatory cytokines, including interleukin-1, interleukin-6 and tumor necrosis factor alpha [12]. Circulating zonulin is also considered to be a potential marker of intestinal permeability in dermatitis herpetiformis [13].

Malyszko et al. [14] assessed serum zonulin levels in kidney transplant recipients. Serum zonulin was significantly lower than in healthy controls. However, zonulin was not related to iron parameters studied. Zonulin concentration was also measured in heart transplant recipients [15] and was significantly lower than in the healthy volunteers. In both studies, zonulin was not related to any of the iron status parameters that is consistent with the results of our study. Authors suggest that low zonulin concentration might occur due to impaired defensive mechanisms caused by immunosuppressive therapy [14, 15].

In our study, a moderate increase in inflammatory markers, typical for CKD, was found. As reported previously by Kotanko et al. [16], degree of systemic inflammation in CKD corresponded to the level of endotoxemia. In sepsis, elevated zonulin levels have been described, probably due to the link between systemic inflammatory response syndrome and increased intestinal permeability [17]. Recently, it has also been reported that higher concentrations of zonulin were associated with the insulin resistance and severity of menstrual disorders in patients with polycystic ovary syndrome (PCO) [18]. Interestingly, in murine models, zonulin has been associated with development of acute lung injury by enhancing albumin leak, complement activation and increased cytokines and neutrophils production [19].

In the recent study by Ficek et al. [20] in hemodialyzed patients, a weak correlation between levels of IL-6 and D–lactates and lipolysaccharide-LPS and the greatest severity of inflammation were found in the highest tertile of zonulin, LPS and D–lactates concentrations, while in multiple regression models, serum hsCRP and plasma IL-6 variability were explained by LPS only. They also stressed that the weak correlations between zonulin and inflammatory parameters suggesting that the other well-known mechanisms of systemic inflammation in HD played a predominant role. Recently, zonulin was assessed in 225 patients carrying automatic implantable cardioverters/defibrillators (AICD) for primary or secondary prevention [21]. The inverse associations between zonulin and creatinine as well as markers of cardiovascular risk (high calprotectin and kynurenine, low homoarginine) were reported. In addition, the authors found that in the subgroup with type 2 diabetes, zonulin increased significantly together with high-sensitivity CRP. Higher circulating zonulin concentrations have been found in subjects with impaired glucose tolerance regardless of body mass [7]. Zonulin was also significantly elevated in patients with type 2 diabetes [5] and associated with inflammatory markers and poor glycemic control [4]. In our studied group, 23 patients were diabetic; however, we did not find statistically significant difference in zonulin levels between diabetic vs non-diabetic subjects (42.9 ng/mL in diabetes vs 36.5 ng/mL in non-diabetes patients with CKD, *p* = 0.09).

There are several limitations of the study. The sample is not large to make definite conclusions, and there is a need for a larger sample and other groups of patients to follow prospectively to conclude whether zonulin as a biomarker will help to distinguish inflammation in CKD. It also should be stressed that our study might be underpowered to show an association and therefore further research are needed to prove or disprove the causality of zonulin and inflammation in early CKD.

Very recently, Vojdani et al. [22] stressed the problem of zonulin variability. Zonulin is a protein the size of 47,000 Da, released from the lamina propria and presented to the submucosal gut immune system [23]. Then, zonulin-specific antibodies are synthesized as an immune response to many environmental factors [23]. According to Vojdani et al. [22], this immune response against zonulin and other large molecules may be an explanation for zonulin fluctuation. As majority of laboratories analyze serum zonulin levels from a single blood draw, variability of serum zonulin levels during the time course of a single day or from day to day should be taken into account. Thus, different studies yielding not comparable results should bear in mind the zonulin variability.

In conclusion, zonulin appears not to be an inflammatory marker in CKD. It seems it also does not play a role in the disturbances of iron metabolism in CKD. Its physiological role remains to be elucidated.
